# Colorectal cancer characterization and therapeutic target prediction based on microRNA expression profile

**DOI:** 10.1038/srep20616

**Published:** 2016-02-08

**Authors:** Peng Xu, Yanliang Zhu, Bo Sun, Zhongdang Xiao

**Affiliations:** 1 State Key Laboratory of Bioelectronics, School of Biological Science and Medical Engineering, Southeast University, Nanjing 210096, P. R. China

## Abstract

Colorectal cancer (CRC) is one of the most commonly diagnosed cancers and a major cause of cancer death. However, the molecular mechanisms underlying CRC initiation, growth and metastasis are poorly understood. In this study, based on our previous work for comprehensively analyzing miRNA sequencing data, we examined a series of colorectal cancer microRNAs expression profiles data. Results show that all these CRC samples share the same four pathways including TGF-beta signaling pathway, which is important in colorectal carcinogenesis. Twenty-one microRNAs that evolved in the four overlapped pathways were then discovered. Further analysis selected miR-21 as an important regulator for CRC through TGF-beta pathways. This study develops methods for discovering tumor specific miRNA cluster as biomarker and for screening new cancer therapy targets based on miRNA sequencing.

Colorectal cancer (CRC) is a major cause of cancer-associated morbidity and mortality all over the world, with an estimated 1.4 million cases and 693,900 deaths occurring in 2012 according to the *Global cancer statistics*^1^,^2^. Invasion and metastasis are the major cause of this deathly cancer type. Although recent improvements in diagnostic techniques and clinical management have increased the early detection rate of CRC and decreased the mortality rate, more than one third of patients with CRC will ultimately develop into metastatic disease^3^. Until now, the pathogenesis of CRC is one of the most characterized carcinogenesis process, while new molecules that play a role in this process are still being discovered.

MicroRNAs (miRNAs) are small(approximately 20 ~ 22 nucleotides), noncoding RNAs that regulate gene expression by the inhibition of the translation and/or decreasing of the stability of their mRNA targets. It has been demonstrated that miRNAs play a significant role in tumorigenesis by down-regulating tumor suppressor genes or oncogenes^4^,^5^. MiRNAs were first discovered in 1993, after that approximately two thousand human miRNAs have been indentified, which have been proved to play important roles in various biological processes, including cell proliferation, cell cycle, apoptosis and differentiation^6^. An increasing number of miRNAs have been observed in various types of cancer and they were considered involving in modulating cancer cell behaviors^7^,^8^. Indeed, dysregulating of miRNAs were proved to be a key process in the pathogenesis of many cancers and may occur at any point, from initiation to metastasis stage^9^,^10^. Many studies have indicated that miRNAs were deregulated in various types of human malignancies such as colon cancer, breast cancer, lung cancer, pancreatic cancer, and chronic lymphocytic leukemia^11^,^12^. Circulating levels of miRNAs are also quite stable and reproducible in the body fluid of many cancer patient. Given the critical role of miRNAs in the development of tumors and their global regulation, it shows great promise to character or screen therapy targets for CRC^13^.

In complex disease analysis for gaining potential delicate biological insights, pathway-based approaches and sub-pathway-based approaches have become the first choice^14^,^15^,^16^. In this study, based on the mathematical model as presented in our previous work^17^, we comprehensively analyze four CRC samples aiming to try to characterize CRC and discover therapeutic targets based on miRNAs profile.

## Materials and Methods

### Data source

The miRNA microarrays that we used in this study were deposited in the National Center for Biotechnology Information (NCBI) Gene Expression Omnibus (GEO) database with accession code GSE35602, which including four cases of CRC samples(GSM871528, GSM871531, GSM871533, GSM871535) and four cases of adjacent normal colorectal samples( GSM871539, GSM871541, GSM871543, GSM871545), as reported in^18^. In this study, total RNAs from epithelial tissue of cancer and normal samples were analyzed by miRNA microarray. Total RNAs were extracted from tissue using the miRNeasyMini Kit (Qiagen) according to the manufacturer’s protocol. Concentrations and purities of the total RNAs were assessed with a spectrophotometer and RNA integrity was verified using an Agilent 2100 Bioanalyzer (Agilent Technologies). Colon adenocarcinoma (COAD) miRNASeq data samples were obtained from The Cancer Genome Atlas (TCGA).

## Methods

### miRNA expression profiles data normalization

Measures of RNA abundance are important for many areas of biology and often obtained from high throughput RNA sequencing methods such as Illumina sequence data. So in this study, when receive miRNA expression profiles data, to remove technical biases inherent in the sequencing approach we first normalize the expression profiles data. Here we employ TPM(Transcripts per million)^19^ method to do this assignment. The TPM of a miRNA *a* is:


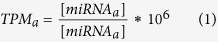



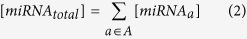


where *A* stands for the set of all miRNAs determined in that experiment and *a* is an index for a miRNA. So both in eq.(1) and (2) , the *miRNA*_*a*_ represents transcripts number of miRNA *a* and *miRNA*_*total*_ represents all miRNAs transcripts number in total in a sample.

### A mathematical model to evaluate the comprehensive repression rate of specific mRNAs using total miRNA expression profiles

The method of evaluating the comprehensive repression rate of specific mRNAs using total miRNA expression profiles is as following^17^:


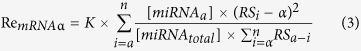


Where *miRNA*_*a*_ and *miRNA*_*total*_ are the same meaning as refer to in above eq.(1) and (2). RS_*i*−*α*_ represents repression score of *miRNA*_*i*_ on their target gene *mRNA*_*α*_, thereinto, *i* represents the indexes of miRNAs that share a specially target gene *mRNA*_*α*_. *RS*_*a*−*i*_ represents repression score of specific *miRNA*_*a*_ on its target gene *miRNA*_*i*_, here, the *i* represents the indexes of mRNAs that *miRNA*_a_ will target on. Such repression score(RS) are extracted from miRanda^20^.

### Statistical analysis

Z-tests were performed to investigate differences in the repression effect of target mRNAs by miRNAs. p0 was calculated using formula (4), which was used to investigate the null hypothesis.


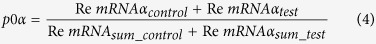


Here, and 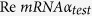 indicates the repression of mRNAα in the control and test groups, whereas 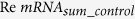 and 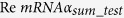 indicates the accumulated repression effect on mRNAs respectively in control and test groups.

Then, the Z-test was performed according to the following formula:


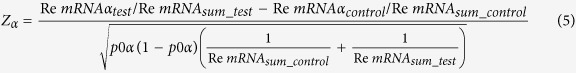


### Method workflow

step 1: Normalization for miRNA expression profile;

step 2: Compute the global repression effect of miRNA expression profile on their target genes;

step 3: Do differential analysis for target genes based on the repression score;

step 4: Do gene function enrichment analysis

## Results and Discussions

Four CRC tissue samples and normal colorectal epithelial tissue datasets were used in this study. The miRNAs expression profiles data for four normal colorectal epithelial were averaged among four samples as control group. Then four samples of CRC were respectively analyzed versus the control group. Currently, the methods for analysing miRNAs profiles are mainly according to the following steps, step 1: differential analysis for miRNAs expression profiles and select the differentially expressed miRNAs; step 2: predicting the targets genes of selected miRNAs; step 3: mapping the selected target genes to whole pathways to find significant enrichment pathways that related to the submitted genes list. However, it has been reported that an individual miRNA may target several mRNAs with different suppression level. The comprehensive repression of a given miRNA pool should be carefully calculated. Indeed, context scores for ranking the predicted targets of each miRNA have been provided by previous studies^21^,^22^,^23^. To comprehensive analyze the global repression effect of miRNAs on their target genes, new method was bring forward in^17^.

**Figure 1:**
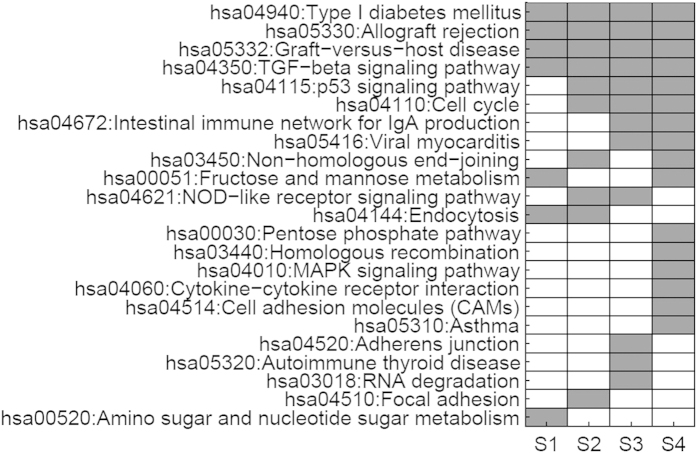
Significantly enriched KEGG pathways of submitted genes that significantly regulated by miRNAs in four CRC samples (S1, S2, S3 and S4). Gray filled block indicates genes are significantly enriched in this pathway in corresponding sample while white filled block indicates genes are not enriched in this pathway.

Four groups of CRC and control samples were analyzed according to the method workflow mentioned in method section. Thereinto gene function enrichment analysis indicates four overlapped KEGG pathways(Type I diabetes mellitus, Allograft rejection, Graft-versus-host disease, TGF-beta signaling pathway)(see Fig. 1).

**Figure 2:**
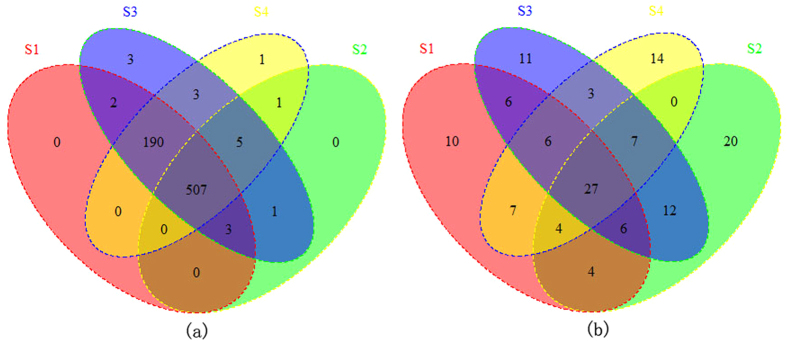
Venn Diagram of miRNAs correlated to the pathways respectively in four samples. All miRNAs that will target to the significantly repressed mRNAs occurring in enriched pathways respectively in four samples (**a**); miRNAs were filtered out after doing differential analysis on the repressed effect for miRNA-mRNA interactions (**b**).

Then, we sort out all the significantly regulated mRNAs that participate in these pathways base on the reference provide by DAVID data base. Due to one miRNA can bind to many mRNAs and one mRNA can also be repressed by multiple miRNAs, hundreds of miRNAs will target to the significantly regulated mRNAs correlated to the overlapped pathways. Quite a few miRNAs (507) were overlapped between the four samples. The Venn Diagram of those mRNAs respectively in four samples of CRC versus control group were plotted in Fig. 2(a). Differential analysis was preformed after getting the repression effect of miRNAs on corresponding mRNAs in enriched pathways. Finally, significant differential miRNA-mRNA interactions were screened out. MiRNAs that occur in the significantly differential miRNA-mRNA interactions were thought to have relatively strong influence and significant differential repressed effect on their corresponding target gene. Results show that 27 miRNAs were overlapped between the four samples as described in Fig. 2(b).

**Figure 3:**
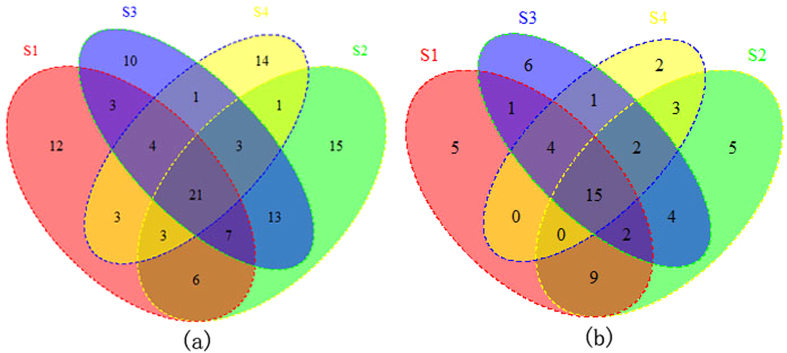
Venn Diagram of miRNAs (**a**) and mRNAs (**b**) correlated to the overlapped four pathways respectively in four samples.

To better understand the potential production mechanism of CRC, we narrow the searching space into four overlapped pathways. By doing differential analysis, results show that 21 miRNAs significantly regulate the mRNAs that correlated to four overlapped pathways as in Fig. 3(a). The mRNAs that were significantly regulated respectively in four samples were also obtained and statistical analysis was done as shown in Fig. 3(b).

For the 21 miRNAs screened out by above differential analysis, most of which also have been reported correlated to pathological mechanism of CRC in previous literatures list as in Table 1. To the best of our knowledge, 3 (3/21, 14.3%) miRNAs namely hsa-miR-16-2*, hsa-miR-30c-2* and hsa-miR-592 are without reports concerning with the CRC. Among the remaining 18 miRNAs , 16 (16/18, 88.9%) are respectively with an consistent change direction and 2 (2/18, 11.1%) with an inconsistent change direction among the published literatures(see Table 1).

**Figure 4:**
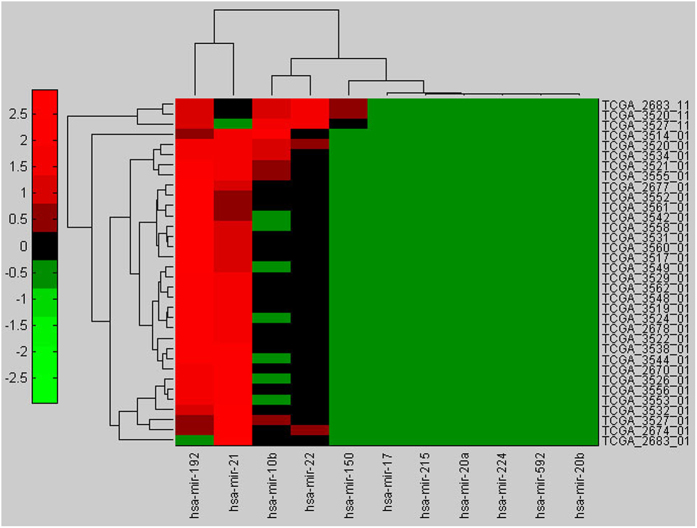
A panel of 11 miRNAs is sufficient to distinguish CRC patients from the healthy subjects. Identifiers for data samples were on the right side of the figure, thereinto, which end with ‘11’ are for healthy subjects and ‘01’ are for CRC samples.

**Figure 5:**
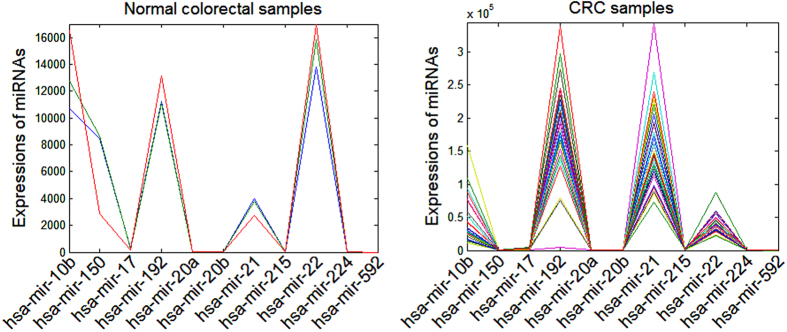
The expression profiles of 11 miRNAs respectively in normal colorectal and CRC samples.

As can be seen from Table 1, most of the 21 miRNAs were validated to be correlated to CRC, which suggests that these miRNAs may be potential biomarkers for CRC diagnosis. To verify this point of view, miRNASeq data samples for COAD stored in TCGA were adopted. Adenocarcinoma of the colon is the most common histopathological type of CRC, here we will uniformly called it as CRC. The miRNASeq data of CRC that we used here were in batch 28 including 31 CRC data samples and 3 matched normal samples. Firstly, we picked up miRNAs sequence data that belong to the 21 miRNAs, 12 miRNAs were selected, thereinto, miR-630 was found to be zero in all the 34 CRC miRNASeq samples. Ignored the miR-630, 11 miRNAs were remained in the data samples. Then, hierarchical clustering analysis in Matlab was adopted on the 34 CRC miRNASeq samples (see Fig. 4). Results show that a panel of 11 miRNAs is sufficient to distinguish CRC patients from the healthy subjects. The expression profiles of 11 miRNAs respectively in normal colorectal and CRC samples were described in Fig. 5. In normal colorectal samples, the expression of miR-21 is lower than miR-192 and miR-22 and expression of miR-22 is slightly higher than that of miR-192. In CRC samples, miR-21 and miR-192 are approximately equivalent in the expression and far more than that of miR-22. Compared with normal colorectal samples, both the expressions of miR-21 and miR-192 are upregulated in CRC samples, but the miR-22 is downregulated.

**Figure 6:**
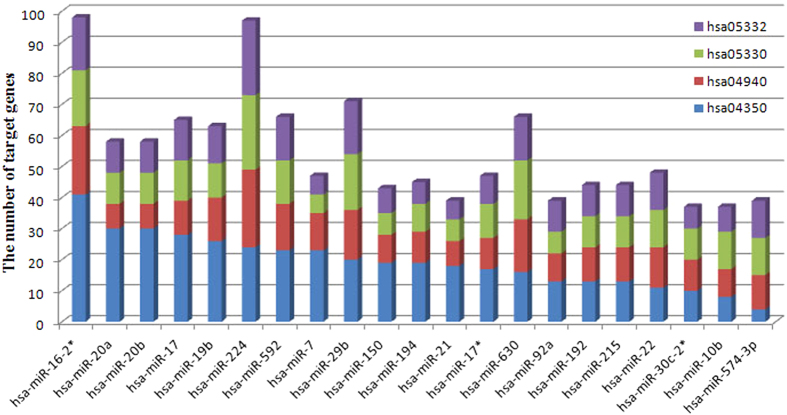
The number of target genes regulated by the 21 miRNAs respectively in the four overlapped pathways (Blue:hsa04350TGF-beta signaling pathway; Red:hsa04940Type I diabetes mellitus; Green: hsa05330Allograft rejection; Purple:hsa05332Graft-versus-host disease).

By KEGG pathway tool^24^, we get the gene lists that evolved in different pathways. Thus, genes that presented in the overlapped four pathways respectively could be selected. Then from the miRNA-mRNA interaction reference given by database such as miRanda^20^, we further get target genes regulated by the 21 miRNAs respectively in the four overlapped pathways (Fig. 6). Among those genes, as can be seen from the Fig. 6, most of those genes evolved in hsa04350TGF-beta signaling pathway. Furthermore, Type I diabetes mellitus, Allograft rejection and Graft-versus-host disease are signaling pathways that correlated to immune response. The reasons for immune response are various. Depending on these immune response pathways, it is difficult to discriminate whether the correlated dysregulated miRNAs are directly related to the CRC or are a secondary response in the histologic progression of the cancer. Bring together these factors, TGF-beta signaling pathway was regarded as the most important pathway that related to the CRC.

**Figure 7:**
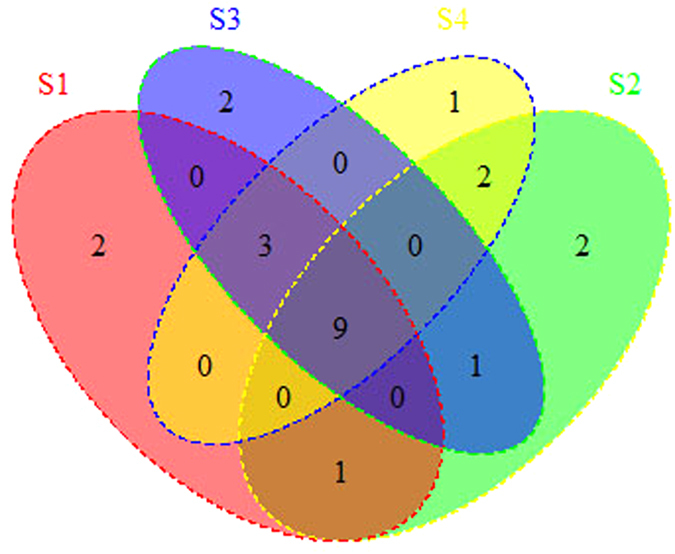
Venn Diagram significantly differential repressed mRNAs in hsa04350TGF-beta signaling pathway respectively in four CRC samples.

**Figure 8:**
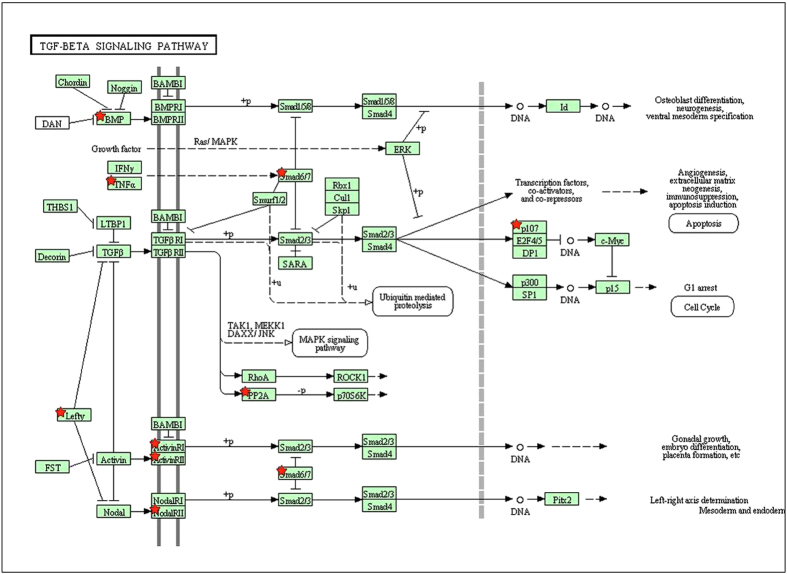
TGF-beta signaling pathway: the display of nine genes on the TGF-beta signaling pathway.

From Fig. 3(b), 15 significantly differential repressed mRNAs were overlapped between the four samples. Next, we paid close attention to the TGF-beta signaling pathway to see the distribution of 15 significantly differential repressed mRNAs between the four samples (see Fig. 7). As can be seen from the diagram, there are nine genes evolved in all the four CRC samples. To study the potential biological function of the nine overlapped genes, we submit only the nine genes and see their distribution on the TGF-beta signaling pathway (see Fig. 8).

TGF-beta signaling pathway plays a unique and pivotal role in homeostasis, wound healing, carcinogenesis, fibrosis, angiogenesis and differentiation of the cell^25^,^26^. TGF-beta and its signaling effectors influence cancer biological behaviors. The TGF-beta signaling pathway was proved to be a double-edged sword in cancer that plays as both a tumor suppressor and a cancer promoter^27^. From the enriched genes in TGF-beta signaling pathway, we can see that genes Smad6/7, which are located in downstream of TNF-alpha, play as an inhibitory factor to Smad2/3. By the activated TGF-βRI receptor, Smad2/3 are phosphorylated at the carboxyl terminal serines and form heteromeric complexes with Smad4. Ultimately, Smad2/3/4 complex translocates into the nucleus and binds to specific regulatory elements on target genes^28^,^29^. Smad 2/3/4 complex once in the nucleus, induces among others, a cyclin-dependent kinase (CDK) inhibitor p21, which will lead to growth arrest^30^. Protein p21 is the product of waf/cip1 gene, an inhibitor of CDK, and is a powerful and reversible inhibitor of the propagation of cell cycle at G1 and G2 activated upon DNA damage. The protein p21 interacts with complexes of CDK2 and cyclin A or cyclin E and thereby inhibits the activity of CDK2, preventing progression of cell cycle^31^.

Indeed, we find that miR-21 has a significantly differential repressed effect on the Smad7 gene and miR-21 is up-regulated in all the four CRC samples respectively (Table in supplementary). In addition, Smad7 has been identified as a target gene of miR-21 namely in fibrotic lung diseases and diabetic nephropathy^32^,^33^. The human miR-21 gene is located on chromosome 17q23-1 overlapping with the TMEM49 gene, a human homologue of rat Vacuole Membrane Protein 1 (VMP1)^34^. There have been several studies indicated that miR-21 expression was significantly higher in CRC compared with the adjacent normal tissues^35^,^36^,^37^,^38^,^39^. Li *et al*. demonstrate that miR-21 and Smad7 are critical regulators of TGF-β1 signaling during the induction of carcinoma associated fibroblasts(CAF) formation^40^. They find that the overexpression of Smad7 or the depletion of miR-21 blocks TGF-β1-induced CAF formation, whereas the overexpression of miR-21 or the depletion of Smad7 promotes CAF formation, even without TGF-β1 stimulation. Carcinoma associated fibroblasts are activated fibroblasts and a key cellular component of tumor stroma. Through specific communication with cancer cells, CAFs not only directly promote tumor progression^41^,^42^ and metastasis^43^,^44^, but are also involved in the initiation of cancer^45^,^46^,^47^. Several studies have reported that the infection of pre-miR-21 lentiviral vector in CRC cell lines including HT-29, Colo206f, LIM 1863, SW480 and DLD1 cells can induce significant cell proliferation, enhance migratory and invasive properties, and decrease apoptosis. In contrast, knockdown of miR-21 could significantly decrease cell proliferation, suppress the invasion, migration and increase the apoptosis^48^,^49^,^50^. Sprouty2 (Spry2) was considered as a tumor suppressor gene in cancer cells. The expression of Spry2 will inhibit the growth of HCT-116 cells, promote apoptosis and induce sensitization to 5-fluorouracil and metformin. But the knockdown of miR-21 in HCT-116 cells correlated with the elevated expression of Spry2, and the proliferation rate of HCT-116 was down-regulated, indicating that function of Spry2 in CRC is regulated by miR-21^51^.

### Validation on wet experiments

**Figure 9:**
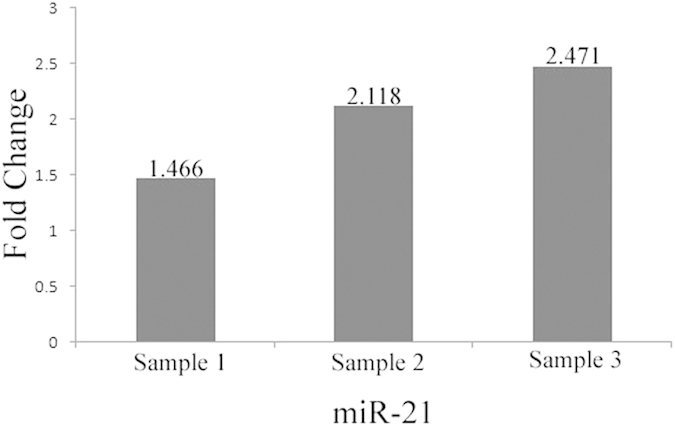
qPCR analysis of miR-21 expression level in three groups of CRC samples versus their corresponding control samples.

It inspirited us that MiR-21 may be regarded as a target for inhibition the outgrowth of CRCs according the above data analysis. To verify this point, some wet experiments were carried out. Three CRC tissue samples and their corresponding paraneoplastic healthy tissue samples were adopted in this section. To dissect the expression difference of miR-21 between CRCs and healthy samples, qPCR analysis was done on the three groups of samples. Results show that the expression level of miR-21 are higher in all cancer samples than that in control groups with fold changes 1.466, 2.118 and 2.471 respectively (see Fig. 9). These results are consistent with the conclusion obtained from above studies and indicates miR-21 could be regarded as a target in CRC treatment.

**Figure 10:**
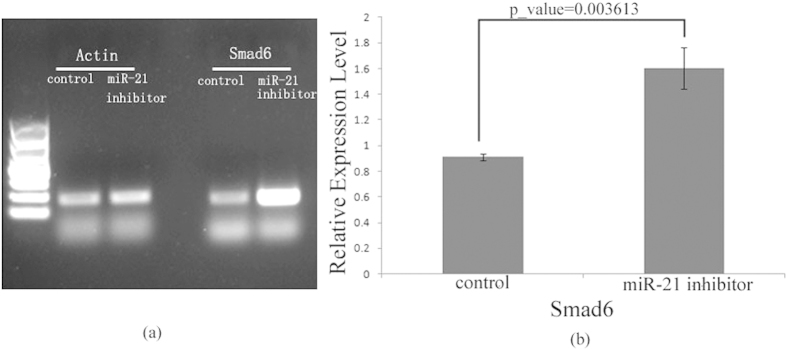
PCR analysis of expression level of Smad6 treated with and without inhibition of miR-21. PCR analysis were performed on Smad6, in which Actin was taken as internal reference (**a**). The same PCR analysis experiment were repeated 4 times, by using of software of imageJ, mean grey level of the PCR results were got and represented as mean ± SD of four independent experiments (p_value = 0.003613) (**b**) .

As described in Fig. 8, differentially repressed Smad6/7 locate in an important position which is correlated to both the cell cycle and embryo differentiation. Smad7 has been validated to be the target gene of miR-21 in previously experiments^32^,^33^ and was regard as a pivot factor in cancer. Interestingly, though both Smad6 and Smad7 are the targets of miR21, there is lack of reports that miR-21 could regulate Smad6 in colon cancer cells, to the best of our knowledge. To examine the Smad6 expression level treated with and without inhibition of miR-21, PCR analysis were implemented. As our expected, results show that the expression level of Smad6 is becoming significantly higher when inhibit the level of miR-21 in colon cancer cells (see Fig. 10).

**Figure 11:**
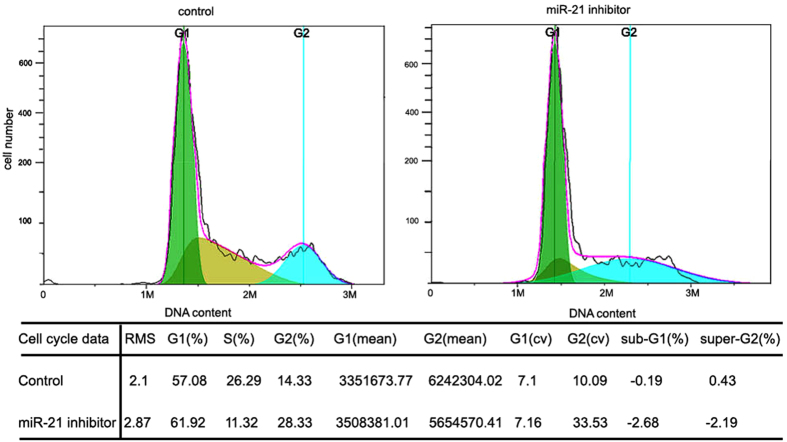
Effects of inhibition of miR-21 on cell cycle distribution in human CRC cell lines.

Finally, to examine the effect of miR-21 on colon cancer cell growth, miR-21 antisense was inducted into the cells, the cell cycle analysis was performed. As our expect, the growth of CRCs was significantly inhibited as shown in Fig. 11. These data suggest miR-21 inhibition in CRCs could effectively impair the growth of tumor cells and thus provide a therapeutic target for CRC treatment.

### Real Time Quantitative Polymerase Chain Reaction (qRT-PCR)

Total RNA of CRC tissue samples and their corresponding paraneoplastic healthy tissue samples were extracted using Trizol reagent according to the supplier’s instruction (Invitrogen, USA) and quantified by spectrophotometry at 260 nm. Reverse transcription was performed for 60 min at 42 °C, followed by 5 min at 85 °C to inactivate the reverse transcriptase. MicroRNA expression level was examined in all samples. The approach of the experiment followed standard protocols, qRT-PCR was performed on the Applied Biosystems 7500 Sequence Detection System (BD, Michigan, USA). The reactions were amplified over 40 cycles of 95 °C for 15s and 60 °C for 1 min. Afterwards, a thermal denaturation protocol was performed to determine the number of products present in each reaction. The reactions were typically run in triplicate. The cycle number at which the reaction crossed an arbitrarily placed threshold (Ct) was determined for each gene. Target gene expression was normalized to the expression of U6 in each sample. Data was analyzed using the 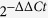 method^52^.

## Conclusions

Due to the stability of miRNAs during analysis and the development of sequencing methods, miRNA expression profiling was identified as a reliable method for investigation of mRNAs, proteins and pathways involved in particular biological processes. In this study, we comprehensively analyze a series of CRC miRNAs expression profiles data, 21 miRNAs that have significantly differential repressed effect on target genes were found. Gene function analysis show that TGF-beta signaling pathway may involved in colorectal carcinogenesis and miR-21 was found to be important in this process. Inhibition miR-21 could hinder the growth of CRC growth. Thus, through miRNA sequencing analyzing, we could characterize the CRC with a panel of 21 miRNAs, as well as select potential target for CRC treatment.

## Additional Information

**How to cite this article**: Xu, P. *et al.* Colorectal cancer characterization and therapeutic target prediction based on microRNA expression profile. *Sci. Rep.* **6**, 20616; doi: 10.1038/srep20616 (2016).

## Supplementary Material

Supplementary Information

## Figures and Tables

**Table 1:** Reports of 21 overlapped miRNAs that significantly regulate the four pathways.

**miRNAs**	**up/down,References**
hsa-miR-17*(hsa-miR-17-3p)	↑^53^.
	↑^54^.
	↑^35^.
hsa-miR-16-2*(hsa-miR-16-2-3p)	NULL
hsa-miR-30c-2*(hsa-miR-30c-2-3p)	NULL
hsa-miR-574-3p	↓^55^.
hsa-miR-592	NULL
hsa-miR-630	↑^56^.
hsa-miR-17	↑^57^.
	↓^58^.
hsa-miR-19b	↑^59^.
	↑^60^.
hsa-miR-21	↑^36^.
	↑^35^.
hsa-miR-22	↓^61^.
hsa-miR-20a	↑^57^.
	↑^59^.
	↑^62^.
	↑^63^.
hsa-miR-92a	↑^64^.
hsa-miR-29b	↑^59^.
hsa-miR-192	↓^57^.
	↓^65^.
hsa-miR-7	↑^66^.
hsa-miR-10b	↓^54^.
	↓^59^.
hsa-miR-215	↓^57^.
	↑^35^.
hsa-miR-224	↑^67^.
	↑^54^.
	↑^59^.
	↑^35^.
hsa-miR-150	↑^63^.
hsa-miR-194	↑^35^.
hsa-miR-20b	↓^54^.
