# Supplementary material for: Colorectal cancer characterization and therapeutic target prediction based on microRNA expression profile
Source: Sci Rep. 2016 Feb 8;6:20616. doi: 10.1038/srep20616 (PMC4745004; doi:10.1038/srep20616)
Supplement: Supplementary Information [file srep20616-s1.pdf]

Colorectal cancer characterization and therapeutic target prediction based on microRNA expression profile

PengXu, Yanliang Zhu, Bo Sun*, Zhongdang Xiao#
State Key Laboratory of Bioelectronics, School of Biological Science and Medical Engineering, Southeast University, Nanjing 210096, P. R. China
Corresponding authors:*sunbo@seu.edu.cn (BS); #zdxiao@seu.edu.cn (ZDX)

S_table 1: CRC092VSControl

#Protein:protein name occurs in the kegg_pathway(miRNA target gene)
#RS_CRC:Repression score of miRNA to target gene in colorectal cancer sample
#RS_control:Repression score of miRNA to target gene in control group
#z_alpha:differential expression variable

Repression effect of miRNA to target gene

miRNA	Protein	RS_CRC	RS_control	P_value
hsa-miR-215	Lefty(LEFTY2)	12.86373	31.78988	0
hsa-miR-192	Lefty(LEFTY2)	22.04721	51.85411	0
hsa-miR-17	TNF-alpha (TNF)	2.335898	0.454436	0.002453208
hsa-miR-19b	TNF-alpha (TNF)	176.0758	47.3179	0
hsa-miR-20a	TNF-alpha (TNF)	6.249487	1.279866	3.57628E-07
hsa-miR-150	TNF-alpha (TNF)	1.661407	6.294316	4.05312E-06
hsa-miR-16-2*	TNF-alpha (TNF)	0.0186221	10.73176	0
hsa-miR-20b	TNF-alpha (TNF)	1.53205	0.3085527	0.01917601
hsa-miR-592	TNF-alpha (TNF)	0.858525	0.005110096	0.01673102
hsa-miR-215	ActivinRI (ACVR1)	35.05997	86.643	0
hsa-miR-192	ActivinRI (ACVR1)	60.08946	141.3279	0
hsa-miR-19b	ActivinRI (ACVR1)	5.679864	1.526384	1.58548E-05
hsa-miR-194	ActivinRI (ACVR1)	17.5203	34.35178	0
hsa-miR-29b	ActivinRI (ACVR1)	6.502964	3.102683	0.003285766
hsa-miR-16-2*	ActivinRI (ACVR1)	0.00801184	4.617154	0
hsa-miR-16-2*	ActivinRII(ACVR2A);	0.02771662	15.97286	0
hsa-miR-630	ActivinRII(ACVR2A);	1.46491	0.03994367	0.001707077
hsa-miR-192	ActivinRII(ACVR2A);	48.84971	114.8924	0
hsa-miR-215	ActivinRII(ACVR2A);	28.50199	70.43639	0
hsa-miR-215	PP2A (PPP2CB)	10.84589	26.80323	0
hsa-miR-192	PP2A (PPP2CB)	18.58883	43.72013	0
hsa-miR-20b	P107(P130)	4.625612	0.9315919	1.20401E-05
hsa-miR-16-2*	P107(P130)	0.00346458	1.996607	9.82285E-05
hsa-miR-17	P107(P130)	7.92174	1.541131	0
hsa-miR-20a	P107(P130)	18.86864	3.864212	0
hsa-miR-92a	P107(P130)	3.091928	0.01029686	8.34465E-07
hsa-miR-92a	SMAD6/7(SMAD7)	24.57269	0.08183295	0
hsa-miR-20a	SMAD6/7(SMAD7)	16.70536	3.421182	0
**hsa-miR-21**	**SMAD6/7(SMAD7)**	**201.7709**	**174.3653**	**9.34601E-05**
hsa-miR-17	SMAD6/7(SMAD7)	7.058474	1.373187	0
hsa-miR-20b	SMAD6/7(SMAD7)	4.095287	0.8247852	4.23193E-05
hsa-miR-16-2*	BMP (BMP5)	0.03875996	22.33704	0

S_table 2: CRC614VSControl

#Protein:protein name occurs in the kegg_pathway(miRNA target gene)
#RS_CRC:Repression score of miRNA to target gene in colorectal cancer sample
#RS_control:Repression score of miRNA to target gene in control group
#z_alpha:differential expression variable

Repression effect of miRNA to target gene				
miRNA	Protein	RS_CRC	RS_control	P_value
hsa-miR-215	Lefty (LEFTY2)	7.685757	26.94696	0
hsa-miR-192	Lefty (LEFTY2)	12.6049	43.95458	0
hsa-miR-150	TNF-alpha (TNF)	1.638092	5.335431	4.399E-05
hsa-miR-592	TNF-alpha (TNF)	2.042412	0.2155119	0.0004795
hsa-miR-16-2*	TNF-alpha (TNF)	0.009881767	9.09687	0
hsa-miR-19b	TNF-alpha (TNF)	79.94386	40.10942	0
hsa-miR-20a	TNF-alpha (TNF)	2.885697	1.08489	0.012871
hsa-miR-194	ActivinRI (ACVR1)	12.63752	29.11858	0
hsa-miR-16-2*	ActivinRI (ACVR1)	0.004251458	3.91377	0
hsa-miR-192	ActivinRI (ACVR1)	34.35452	119.7978	0
hsa-miR-29b	ActivinRI (ACVR1)	7.843195	2.630016	1.907E-06
hsa-miR-215	ActivinRI (ACVR1)	20.94745	73.44368	0
hsa-miR-215	ActivinRII(ACVR2A);[illegible]	17.02923	59.70601	0
hsa-miR-192	ActivinRII(ACVR2A);[illegible]	27.9285	97.38956	0
hsa-miR-16-2*	ActivinRII(ACVR2A);[illegible]	0.01470775	13.53953	0
hsa-miR-630	ActivinRII(ACVR2A);[illegible]	3.894553	1.684574	0.0095972
hsa-miR-192	PP2A (PPP2CB)	10.62766	37.05975	0
hsa-miR-215	PP2A (PPP2CB)	6.480148	22.71999	0
hsa-miR-17	P107(P130)	3.384997	1.306353	0.0076424
hsa-miR-20a	P107(P130)	8.712585	3.275532	3.576E-06
hsa-miR-20b	P107(P130)	2.101533	0.7896718	0.0392389
hsa-miR-16-2*	P107(P130)	0.001838468	1.692441	0.0001704
hsa-miR-20a	SMAD6/7(SMAD7)	7.713691	2.899994	1.454E-05
**hsa-miR-21**	**SMAD6/7(SMAD7)**	**193.8606**	**147.8022**	**0**
hsa-miR-92a	SMAD6/7(SMAD7)	9.982863	3.451202	1.192E-07
hsa-miR-17	SMAD6/7(SMAD7)	3.016119	1.163994	0.0126216
hsa-miR-16-2*	BMP (BMP5)	0.02056786	18.93419	0

S_table 3: CRC870VSControl

#Protein:protein name occurs in the kegg_pathway(miRNA target gene)
#RS_CRC:Repression score of miRNA to target gene in colorectal cancer sample
#RS_control:Repression score of miRNA to target gene in control group
#z_alpha:differential expression variable

Repression effect of miRNA to target gene

miRNA	Protein	RS_CRC	RS_control	P_value
hsa-miR-215	Lefty (LEFTY2)	12.2345	26.94696	0
hsa-miR-192	Lefty (LEFTY2)	21.05387	43.95458	0
hsa-miR-19b	TNF-alpha (TNF)	94.20999	40.10942	0
hsa-miR-17	TNF-alpha (TNF)	1.620653	0.3852065	0.005755186
hsa-miR-20a	TNF-alpha (TNF)	3.778829	1.08489	5.51939E-05
hsa-miR-150	TNF-alpha (TNF)	1.506696	5.335431	8.34465E-07
hsa-miR-16-2*	TNF-alpha (TNF)	0.0117057	9.09687	0
hsa-miR-592	TNF-alpha (TNF)	1.820211	0.2155119	0.000233293
hsa-miR-215	ActivinRI (ACVR1)	33.34501	73.44368	0
hsa-miR-192	ActivinRI (ACVR1)	57.38213	119.7978	0
hsa-miR-19b	ActivinRI (ACVR1)	3.039032	1.293852	0.008340001
hsa-miR-194	ActivinRI (ACVR1)	16.54158	29.11858	0
hsa-miR-29b	ActivinRI (ACVR1)	5.284353	2.630016	0.002523303
hsa-miR-16-2*	ActivinRI (ACVR1)	0.0050362	3.91377	0
hsa-miR-630	ActivinRII(ACVR2A);NodalRII	0.6073588	1.684574	0.02953291
hsa-miR-16-2*	ActivinRII(ACVR2A);NodalRII	0.0174224	13.53953	0
hsa-miR-192	ActivinRII(ACVR2A);NodalRII	46.64878	97.38956	0
hsa-miR-215	ActivinRII(ACVR2A);NodalRII	27.10781	59.70601	0
hsa-miR-215	PP2A (PPP2CB)	10.31536	22.71999	0
hsa-miR-192	PP2A (PPP2CB)	17.7513	37.05975	0
hsa-miR-17	P107(P130)	5.496128	1.306353	0
hsa-miR-20a	P107(P130)	11.40916	3.275532	0
hsa-miR-16-2*	P107(P130)	0.0021778	1.692441	1.62125E-05
hsa-miR-20b	P107(P130)	2.802547	0.7896718	0.000556707
hsa-miR-20b	SMAD6/7(SMAD7)	2.481236	0.6991362	0.001266122
hsa-miR-92a	SMAD6/7(SMAD7)	10.07223	3.451202	0
hsa-miR-20a	SMAD6/7(SMAD7)	10.1011	2.899994	0
**hsa-miR-21**	**SMAD6/7(SMAD7)**	**199.9805**	**147.8022**	**0**
hsa-miR-17	SMAD6/7(SMAD7)	4.897191	1.163994	3.57628E-07
hsa-miR-16-2*	BMP (BMP5)	0.0243641	18.93419	0

S_table 4: CRC936VSControl

#Protein:protein name occurs in the kegg_pathway(miRNA target gene)
#RS_CRC:Repression score of miRNA to target gene in colorectal cancer sample
#RS_control:Repression score of miRNA to target gene in control group
#z_alpha:differential expression variable

Repression effect of miRNA to target gene

miRNA	Protein	RS_CRC	RS_control	P_value
hsa-miR-215	Lefty （LEFTY2）	6.657634	26.94696	0
hsa-miR-192	Lefty （LEFTY2）	11.20894	43.95458	0
hsa-miR-17	TNF-alpha （TNF）	1.711352	0.3852065	0.017182
hsa-miR-19b	TNF-alpha （TNF）	116.4342	40.10942	0
hsa-miR-20a	TNF-alpha （TNF）	4.291719	1.08489	0.000152
hsa-miR-150	TNF-alpha （TNF）	0.8659856	5.335431	4.77E-07
hsa-miR-592	TNF-alpha （TNF）	1.949178	0.2155119	0.001511
hsa-miR-16-2*	TNF-alpha （TNF）	0.0170961	9.09687	0
hsa-miR-192	ActivinRI （ACVR1）	30.54985	119.7978	0
hsa-miR-29b	ActivinRI （ACVR1）	10.26926	2.630016	0
hsa-miR-19b	ActivinRI （ACVR1）	3.755943	1.293852	0.003459
hsa-miR-215	ActivinRI （ACVR1）	18.14532	73.44368	0
hsa-miR-194	ActivinRI （ACVR1）	9.070271	29.11858	0
hsa-miR-16-2*	ActivinRI （ACVR1）	0.0073553	3.91377	0
hsa-miR-16-2*	ActivinRII(ACVR2A);Noda	0.0254453	13.53953	0
hsa-miR-630	ActivinRII(ACVR2A);Noda	0.3736494	1.684574	0.017482
hsa-miR-215	ActivinRII(ACVR2A);Noda	14.75123	59.70601	0
hsa-miR-192	ActivinRII(ACVR2A);Noda	24.83549	97.38956	0
hsa-miR-192	PP2A （PPP2CB）	9.450674	37.05975	0
hsa-miR-215	PP2A （PPP2CB）	5.613299	22.71999	0
hsa-miR-20b	P107(P130)	3.134314	0.7896718	0.001431
hsa-miR-92a	P107(P130)	1.544399	0.4342572	0.04532
hsa-miR-17	P107(P130)	5.803717	1.306353	2.62E-06
hsa-miR-20a	P107(P130)	12.95769	3.275532	0
hsa-miR-16-2*	P107(P130)	0.0031807	1.692441	0.000415
hsa-miR-20a	SMAD6/7(SMAD7)	11.4721	2.899994	0
**hsa-miR-21**	**SMAD6/7(SMAD7)**	**235.1401**	**147.8022**	**0**
hsa-miR-17	SMAD6/7(SMAD7)	5.171261	1.163994	1E-05
hsa-miR-92a	SMAD6/7(SMAD7)	12.27391	3.451202	0
hsa-miR-20b	SMAD6/7(SMAD7)	2.774966	0.6991362	0.002901
hsa-miR-16-2*	BMP （BMP5）	0.0355836	18.93419	0
